# Joyce Wamoyi: changing behaviours for better health

**DOI:** 10.2471/BLT.21.031121

**Published:** 2021-11-01

## Abstract

Joyce Wamoyi talks to Andréia Azevedo Soares about the need for long-term, comprehensive approaches to behaviour change initiatives in sub-Saharan Africa.

*Q: How did you become interested in the behavioural aspects of SRH?*

A: When I finished my bachelor’s in 1998, I got a job as a research assistant and started working on issues related to reproductive health and risk for adolescents. I was doing qualitative research using an ethnographic approach (research in which researchers live, observe and/or interact with a study's participants in their real-life environment) which led me to spend prolonged periods in poor communities. I became fascinated by the lives of adolescent girls – what their aspirations were, how they were treated and what the underlying drivers of different behavioural phenomena were, notably behaviours relating to sex. That’s how I became interested in SRH and, in particular, the issue of transactional sex.

*Q: What is traded in transactional sex?*

A: It varies, from basic commodities such as food, shelter, clothing, to more complex items, including intangibles such as social status. In most situations, several commodities are in play, while some commodities may meet several needs. To take a relatively simple example, an adolescent or young woman might exchange sex for a mobile phone, a commodity which meets a concrete need for connectivity but which may also meet the need or desire to be seen with such a phone, signalling higher social status or power. Consideration of these different aspects is very important when designing public health interventions aimed at changing behaviours. Simple interventions such as cash transfer programmes may have an impact in a community of women offering sex to meet basic needs but may be less effective in more complex socioeconomic contexts where motivations may also be complex. Generally, I would say that the programmes implemented in low-income settings that focus solely on alleviating poverty struggle to achieve their goals. Providing people with three or four dollars every quarter may be helpful, but it may not meaningfully change the way adolescent or young woman behave.

*Q: How are cash transfers best targeted to bring about behaviour change?*

A: Cash transfers have been shown to be beneficial in some settings where they encourage specific behaviours: for example, conditional and unconditional cash transfers encouraging women to access health services or attend school. The effectiveness of such interventions has been demonstrated in Malawi, for example, where cash transfers conditional on female school attendance reduced the prevalence of HIV and other sexually transmitted infections. There have also been some programmes in different countries in sub-Saharan Africa designed to increase business opportunities for young women and to teach girls how to manage their, often, limited resources. However, qualitative research suggests that micro-credit interventions have had only a limited impact on transactional sex and may actually increase its prevalence by encouraging young women into the marketplace, a male-dominated space in most countries. The bottom line is that simply focusing on one structural driver – such as poverty, for example – is not generally effective. That is why the more comprehensive programmes have tended to have more of an impact.

*Q: Can you give an example?*

A: The DREAMS (Determined, Resilient, Empowered, AIDS-free, Mentored, and Safe) Partnership initiative is a good example. It was launched in 2014/2015 by PEPFAR (the United States President’s Emergency Plan for AIDS Relief) with private-sector partners and has been implemented in 10 countries so far. DREAMS employs a comprehensive, multisectoral approach that addresses many different factors increasing young women’s exposure to sexual risk, including structural factors such as a lack of access to secondary school, exclusion from economic opportunities and gender-based violence. Delivering multiple interventions at the same time, DREAMS comprises biomedical, structural and behavioural interventions and is credited not only with significantly reducing new HIV diagnoses among adolescent girls and young women, but also with encouraging partner governments to renew their commitments on gender equality and adolescent health and development.

Another good example is the Stepping Stones initiative that was implemented in rural South Africa and focused on addressing harmful gender norms, especially those related to masculinity. Basically, Stepping Stones sought to encourage gender-equitable relationships with better communication between partners. A randomized controlled trial of school student participants in Stepping Stones found a significant reduction in women’s and men’s herpes simplex virus acquisition and a reduction in men’s self-reported interpersonal violence perpetration.

Such programmes show that there is considerable scope for behavioural interventions as a part of more comprehensive initiatives. However, for health benefits to be fully realized, they need to be supported over the long term. The Stepping Stones programme only ran from 2003 to 2006, which is not long enough to impact the structural drivers of risk such as poverty or to bring about change in sexual and social norms that entail exposure to infection, including, of course, condom use.

*Q: Can you say more about that?*

Sexual norms in Tanzania and in many African settings tend to support male control of what happens in sexual relationships, including how often and when sex can occur, whether or not a condom should be used, be it for protection or family planning use, etc. Unequal gender power is a major obstacle to women being able to negotiate condom and contraception use. As a result, they are exposed not only to infection but also to unwanted or unplanned pregnancies. However, once again, interventions that focus solely on or prioritize pre-exposure prophylaxis and condom use may fail to yield the required outcomes for women’s health and well-being. Women need to be empowered outside the bedroom as well. We should not want only to reduce the HIV exposure among adolescent girls but should also be working to improve their well-being and help them to achieve brighter futures. This requires collectively tackling underlying determinants such as poverty, lack of access to education and economic opportunity, and repressive, risky gender norms. Tackling those different areas requires multisectoral interventions.

“More comprehensive programmes have tended to have more of an impact.”

*Q: Is sustaining multisectoral interventions over the long term financially feasible?*

A: This is a very good question, and it begs another. Who decides? Governments? Donors? In both cases there is a tendency to focus on single issues, rather than addressing the broader drivers of morbidity and mortality and to set deadlines for the achievement of targets. This is particularly problematic when we are trying to tackle the structural drivers of risk because, in fact, a robust structural intervention may take many years and has to be implemented on many fronts, combining, for example, provision of condoms, cash transfers and encouraging reflection regarding gender norms and parenting more broadly.

There is a natural tendency for donors to tackle single issues. There is a corresponding tendency among the community bidding for funding to focus on those issues, rather than tackling broader concerns that are less easily defined, monitored and measured. That is where I think governance should play more of a role. Evidence-based institutions, whether national or global, should be able to set priorities and call for resources based on need rather than having to accept narrowly focused responses to some isolated challenge or situation.

*Q: How has COVID-19 impacted sexual and reproductive health-relevant behaviours, including transactional sex, in Tanzania?*

A: There is a need for more research to explore the impact of COVID-19 on transactional sex prevalence, but it seems likely that the social and economic conditions created by COVID will have made a difference. For example, many parents and caregivers have experienced some degree of economic hardship during the pandemic and may, thus, be less able to provide for the basic needs of children. As a result, adolescent girls and young women may be experiencing pressure to engage in transactional sex to meet their different needs. More broadly speaking, since the death of the president in March 2021, people have become increasingly educated about COVID-19, including the infection risks it represents, and may thus be foregoing health services, including SRH services, due to a heightened perception of infection risks.

*Q: Going forward, what do you consider the main challenges in regard to SRH in Tanzania?*

A: Clearly the pandemic itself is going to be a challenge for some time, notably in regard to its impact on the delivery of and access to essential health services. I am talking about SRH services, including HIV prevention services. Beyond that, the main challenge is mobilizing the resources required to address the structural drivers of SRH risk for adolescent and young women. At the very least this will mean tackling disparities in access to education and economic opportunity, while also addressing harmful cultural norms. In other words, it is going to require a concerted, long-term effort across a range of sectors rather than narrowly focused, short-term interventions.

